# Low Expression of 14-3-3beta Is Associated With Adverse Survival of Diffuse Large B-Cell Lymphoma Patients

**DOI:** 10.3389/fmed.2019.00237

**Published:** 2019-10-30

**Authors:** Chaoping Li, Zhaoming Li, Mingzhi Zhang

**Affiliations:** ^1^Department of Oncology, The First Affiliated Hospital of Zhengzhou University, Zhengzhou, China; ^2^Academy of Medical Science of Zhengzhou University, Zhengzhou, China

**Keywords:** 14-3-3beta, diffuse large B-cell lymphoma (DLBCL), prognosis, immunohistochemistry, Kaplan-Meier analysis

## Abstract

Diffuse large B-cell lymphoma (DLBCL), the most common type of non-Hodgkin's lymphoma in the world, is highly heterogeneous. Although current therapies have improved the clinical outcome, 30–40% of the patients are still not cured. Thus, novel treatment options such as targeted therapy is urgently needed. Accumulating evidence suggests that 14-3-3beta protein plays an important role in tumorigenesis and tumor progression. However, the specific roles of 14-3-3beta in DLBCL are still poorly understood. In this study, we retrospectively analyzed 120 archived wax blocks obtained from patients with DLBCL (*n* = 70) and non-neoplastic lymph nodes (*n* = 50). Immunohistochemical staining showed that 14-3-3beta gene expression was significantly decreased in DLBCL tissues (*P* < 0.001) compared to that in non-neoplastic lymph nodes. Low 14-3-3beta expression was significantly correlated with extra-nodal status (*P* = 0.026), serum LDH level (*P* = 0.023) and adverse survival of DLBCL patients. In survival analyses, low 14-3-3beta expression was significantly associated with adverse overall survival of the DLBCL patients (*P* = 0.003). Using the Kaplan-Meier analysis module of the R2 microarray analysis and visualization platform (http://r2.amc.nl), we also confirmed that low expression of 14-3-3beta gene had inferior overall survival in DLBCL patients. Based on our results, we conclude that low expression of 14-3-3beta is associated with adverse survival of diffuse large B-cell lymphoma patients, suggesting a novel prognostic marker and potential therapeutic target.

## Introduction

Diffuse large B-cell lymphoma (DLBCL) is the most common subtype of adult non-Hodgkin's lymphoma, and its incidence has been increasing in recent years, accounting for ~30% of all cases of non-Hodgkin's lymphoma in Western countries (1) and up to 45.8% in China (2). DLBCL shows high heterogeneity in terms of clinical manifestations, genetic findings, treatment response, and prognosis. Although the widespread use of rituximab in combination with chemotherapy has resulted in a significant increase in the therapeutic response rate, a significant proportion of DLBCL shows strong invasive characteristics and is not sensitive to first-line treatment, often resulting in early recurrence. Therefore, strategies for prolonging the recurrence period and/or survival of refractory DLBCL patients and improving the overall prognosis has become a hot research topic.

The 14-3-3beta gene, also known as YWHAB, official full name is tyrosine 3-monooxygenase/tryptophan 5-monooxygenase activation protein beta, a gene encoding 14-3-3beta protein, is located on chromosome 20q13.1 with 8 exons. The 14-3-3beta protein belongs to the highly conserved 14-3-3 protein family found widely from plants to mammals. Accumulating evidence suggests that 14-3-3beta protein plays an important role in tumorigenesis and tumor progression. For example, increased expression of 14-3-3beta has been observed in various types of solid tumors, including vulvar carcinoma (3), lung cancer (4), astrocytoma (5), gliom (6, 7), squamous cell carcinoma (3), colorectal cancer (8), gastric cancer (9), and hepatocellular carcinoma (10). Furthermore, Chien et al. (9) reported that 14-3-3beta expression was significantly upregulated in untreated gastric cancer cells. Overall, these studies support the possibility of YWHAB acting as an oncogene. However, the specific roles of 14-3-3beta in DLBCL are still poorly understood. Therefore, we used immunohistochemistry to compare the expression of 14-3-3beta in tissue samples from DLBCL patients and reactive hyperplasia of lymph nodes from control subjects. Interestingly, we found that the expression of 14-3-3beta gene was decreased in DLBCL patients. In the current study, we explored the potential association of 14-3-3beta protein expression in DLBCL with clinical outcomes. These findings may provide new insight into the pathogenic mechanisms of DLBCL and suggest novel treatment strategies.

## Materials and Methods

### Clinical Data

We retrospectively analyzed 120 archived wax blocks obtained from tissues of patients at the Department of Pathology, the First Affiliated Hospital of Zhengzhou University from January 2007 to December 2012, including 70 DLBCL samples and 50 non-neoplastic lymph node samples, all above were diagnosed by pathology. The DLBCL cohort included 37 males (53.0%) and 33 females (47.0%), aged 13–76 years, with a median age of 49.5 years. The control group included 50 patients with reactive hyperplasia of lymph nodes, 23 females and 27 males, aged 15–65 years, with a median age of 43 years. This study was approved by the Research Ethics Committee of the First Affiliated Hospital of Zhengzhou University. All experiments were performed in accordance with approved guidelines and regulations. Following the Declaration of Helsinki, all participants were patients of the First Affiliated Hospital of Zhengzhou University (Henan, China) and provided signed informed consent prior to their inclusion in the study.

### Histological and Immunohistochemical Testing

All tissue samples were fixed in formaldehyde and embedded in paraffin using standard methods (11). The tissues were then cut into 3–4-μm-thick sections and placed on superfrost-charged glass microscope slides. The slides were deparaffinized in xylene, and rehydrated in a graded ethanol series. The antigens were retrieved in 0.01 M sodium citrate buffer (pH 6.0) using a microwave oven, and 3% hydrogen peroxide was used to block endogenous peroxidase activity. After washing the slides in phosphate-buffered saline with 10% bovine serum albumin for 60 min to prevent non-specific binding, they were incubated with the primary antibodies against 14-3-3beta (YWHAB) (ab97273; 1:100), CD10 (56C6; 1:50), BCL6 (ab41845; 1:50), and MUM-1 (EPR5699; 1:100) (all from Abcam, Cambridge, UK) overnight at 4°C. The next day, the tissues were incubated with horseradish peroxidase-labeled goat anti-rabbit and anti-mouse secondary antibodies (1:1,000; Zhongshan Jinqiao Biological Technology Co., Ltd., Beijing, China) for 60 min at room temperature. Immunostaining was carried out using a DAB substrate kit (Zhongshan Jinqiao Biological Technology Co., Ltd., Beijing, China), followed by immersing in hematoxylin for nuclear counterstaining.

The staining index was obtained based on the scores for the intensity of positive staining (negative, 0; weak, 1; moderate, 2; strong, 3) and the proportion of immunopositive cells of interest (0–5%, 0; <25%, 1; 25–50%, 2; >50–75%, 3; ≥75%, 4) (12). The final score was obtained by multiplying the percentage of positive cells and the staining intensity score for each specimen. All specimens were subdivided according to their overall scores: absent expression (–), 0 points; weak expression (+), 1–4 points; moderate expression (++), 5–8 points; strong expression (+++), 9–12 points. According to the overall scores, the study cohort divided into two categories: (–), (+), were considered to be 14-3-3beta low expression (<5 points) and (++), (+++) were high expression (≥5 points). According to Han's algorithm (13), cases were assigned to the germinal center B-cell like (GCB) DLBCL group based on positive results for CD10 or to the non-germinal center B-cell like DLBCL group (non-GCB) based on negative results for CD10 and BCL6.

All samples were anonymized and independently scored by two investigators, without prior knowledge of the patient characteristics. In case of disagreement, the sample was re-examined until a final consensus was reached.

### Statistical Analysis

Data were analyzed using the Statistical Package for the Social Sciences (SPSS version 21, Chicago, IL, USA), and the counting data were evaluated with the χ^2^ test. Overall survival (OS) was assessed according to the Kaplan-Meier method, and the log-rank test was used for comparisons. Univariate analysis of the association of 14-3-3beta expression with survival was performed using the Kaplan-Meier analysis module of the R2 microarray analysis and visualization platform (http://r2.amc.nl). *p*-values < 0.05 were considered statistically significant.

## Results

### Patient Characteristics

The clinical parameters of the 70 patients with DLBCL are summarized in Table 1. The DLBCL cohort included 37 males (53.0%) and 33 females (47.0%), aged 13–76 years, with a median age of 49 years. The control group included 50 patients with reactive hyperplasia of lymph nodes, 23 females and 27 males, aged 15–65 years, with a median age of 43 years. As shown in Table 2, according to the immunostaining of CD10, BCL6 and MUM1, patients can be divided into GCB and Non-GCB.

**Table 1:** Relationship between the expression of 14-3-3beta (YWHAB) and clinical parameters of DLBCL patients.

**Parameter**	***N***	**Expression of 14-3-3beta**		**χ^**2**^**	***P***
		**Low (*N*)**	**High (*N*)**		
**Age**					
≥60	19	15	4		
<60	51	34	17	0.994	0.319
**Gender**					
Male	37	24	13		
Female	33	25	8	0.986	0.321
**Extra-nodal status**					
<2	15	7	8		
≥2	55	42	13	4.949	0.026^*^
**GC/NGC**					
NGC	38	30	8		
GC	32	19	13	3.169	0.075
**Serum LDH level**					
<300	29	16	13		
≥300	41	33	8	5.184	0.023^*^
**Clinical stage**					
I-II	27	15	12		
III-IV	43	34	19	0.557	0.456
**IPI score**					
0-2	33	20	13		
≥3	37	29	8	2.264	0.105

*LDH, lactate dehydrogenase; IPI, International Prognostic Index; NGCB, non-germinal center B-cell; GCB, germinal center B-cell*.

* *P < 0.05*.

**Table 2:** Immunohistochemical results of the DLBCL patient cohort (*n* = 70).

	**Age**	**Gender**	**BCL6**	**MUM1**	**CD10**	**CD20**	**Bcl-2**	**CD30**	**CD79a**	**GC/Non-GC**
1	44	Male	–	–	–	+	+	+	+	Non-GC
2	68	Male	–	–	–	+	+	+	+	Non-GC
3	39	Female	–	–	–	+	+	+	+	Non-GC
4	71	Male	–	–	–	+	+	+	+	Non-GC
5	50	Male	–	+	–	+	+	–	+	Non-GC
6	48	Male	–	–	–	+	+	–	+	Non-GC
7	37	Male	–	–	–	+	+	+	+	Non-GC
8	63	Female	–	+	–	+	+	+	+	Non-GC
9	62	Male	–	–	–	+	+	+	+	Non-GC
10	41	Female	–	–	–	+	+	+	+	Non-GC
11	37	Female	–	–	–	+	+	–	+	Non-GC
12	59	Male	–	–	–	+	+	+	+	Non-GC
13	27	Female	–	–	–	+	–	–	+	Non-GC
14	34	Male	–	–	–	+	+	+	+	Non-GC
15	40	Female	–	+	–	+	+	–	+	Non-GC
16	20	Female	–	–	–	+	+	+	+	Non-GC
17	69	Male	–	–	–	+	+	–	+	Non-GC
18	38	Male	–	+	–	+	+	+	+	Non-GC
19	59	Female	–	–	–	+	+	–	+	Non-GC
20	35	Female	–	–	–	+	+	+	+	Non-GC
21	47	Male	–	–	–	+	+	+	+	Non-GC
22	33	Male	–	–	–	+	+	+	+	Non-GC
23	18	Female	–	–	–	+	–	–	+	Non-GC
24	62	Male	–	–	–	+	–	–	+	Non-GC
25	66	Male	–	–	–	+	–	+	+	Non-GC
26	56	Male	–	–	–	+	+	+	+	Non-GC
27	57	Male	–	–	–	+	+	–	+	Non-GC
28	52	Female	–	–	–	+	+	+	+	Non-GC
29	56	Male	–	–	–	+	+	+	+	Non-GC
30	44	Female	–	–	–	+	+	–	+	Non-GC
31	58	Male	–	–	–	+	+	–	+	Non-GC
32	27	Male	–	–	–	+	+	–	+	Non-GC
33	36	Male	–	–	–	+	+	+	+	Non-GC
34	46	Female	–	–	–	+	+	+	+	Non-GC
35	45	Male	–	–	–	+	+	–	+	Non-GC
36	54	Male	–	+	–	+	+	–	+	Non-GC
37	52	Female	–	–	–	+	+	–	+	Non-GC
38	60	Female	–	–	–	+	+	+	+	Non-GC
39	58	Male	+	–	+	+	+	–	+	GC
40	76	Female	+	–	–	+	+	–	+	GC
41	75	Male	–	–	+	+	+	–	+	GC
42	31	Female	+	–	–	+	+	–	+	GC
43	13	Male	+	–	+	+	–	+	+	GC
44	41	Female	–	–	+	+	–	+	+	GC
45	53	Female	–	–	+	+	–	+	+	GC
46	60	Female	+	–	–	+	+	+	+	GC
47	40	Female	–	–	+	+	+	+	+	GC
48	34	Male	–	–	+	+	+	+	+	GC
49	40	Male	–	–	+	+	+	–	+	GC
50	56	Male	+	–	–	+	+	–	+	GC
51	69	Female	–	–	+	+	+	+	+	GC
52	64	Female	–	–	+	+	–	+	+	GC
53	45	Male	–	–	+	+	–	–	+	GC
54	62	Female	+	–	–	+	+	–	+	GC
55	34	Female	–	–	+	+	+	+	+	GC
56	37	Female	+	–	–	+	+	+	+	GC
57	46	Female	+	–	–	+	+	–	+	GC
58	56	Male	+	–	–	+	+	–	+	GC
59	49	Female	–	+	+	+	+	–	+	GC
60	44	Female	–	+	+	+	+	–	+	GC
61	38	Female	–	+	+	+	+	+	+	GC
62	65	Male	+	–	–	+	+	+	+	GC
63	44	Female	+	–	–	+	–	+	+	GC
64	51	Male	+	–	–	+	+/–	+	+	GC
65	65	Male	+	–	–	+	–	–	+	GC
66	76	Female	+	–	–	+	–	+	+	GC
67	44	Male	+	–	–	+	+/–	–	+	GC
68	64	Male	–	–	+	+	–	–	+	GC
69	62	Male	+	–	–	+	+/–	–	+	GC
70	59	Female	+	–	–	+	+	–	+	GC

*Non-GC, non-germinal center B-cell; GC, germinal center B-cell*.

### Low Expression of 14-3-3beta in DLBCL

**Figure 1:**
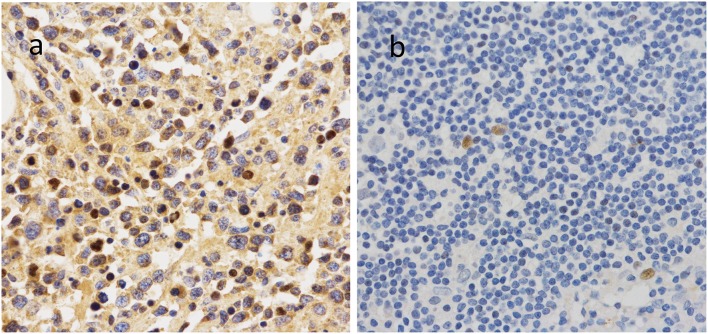
Expression of 14-3-3beta protein in non-neoplastic lymph nodes **(a)** and DLBCL tissues **(b)**.

**Figure 2:**
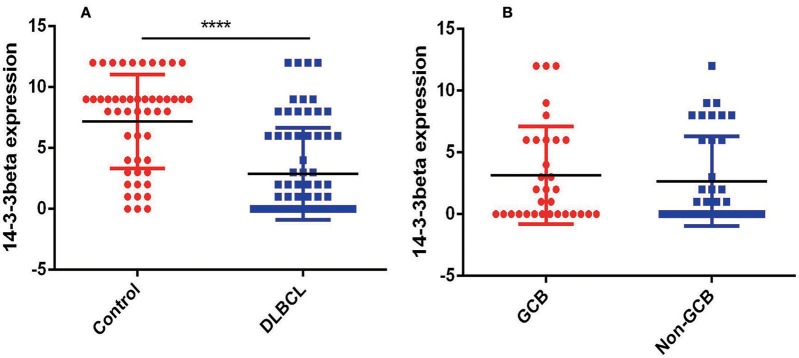
Comparison of 14-3-3 protein expression. **(A)** Expression in non-neoplastic lymph nodes and DLBCL tissues (****p < 0.0001). **(B)** Expression in GCB and Non-GCB DLBCL tissues (*p* = 0.5855).

To further investigate the clinical significance of 14-3-3beta in DLBCL, the correlation between 14-3-3beta expression and clinicopathological features was analyzed (Table 1). Immunohistochemistry (IHC) showed that positive 14-3-3beta expression occurred mostly in the cytoplasm, with weaker expression in the nucleus in the DLBCL group, whereas this pattern was reversed in the non-neoplastic lymph nodes (Figure 1). Moreover, 30% (21/70) and 70% (49/70) of the DLBCL tissues showed high and low 14-3-3beta expression, respectively. In contrast, the majority of non-neoplastic lymph nodes tissues showed high expression (70%, 35/50), with only 30% (15/50) showing low expression. Therefore, we can conclude that 14-3-3beta protein expression is downregulated in DLBCL tissues compared with corresponding levels in non-neoplastic lymph nodes. These differences between groups were statistically significant (*p* < 0.0001; Figure 2A). In addition, we also observed the expression of 14-3-3beta in GCB and Non-GCB DLBCL and found the 14-3-3beta expression was not statistically significant between the two groups (*p* = 0.5855; Figure 2B).

### Low Expression of 14-3-3beta Predicted Poor OS in DLBCL

**Figure 3:**
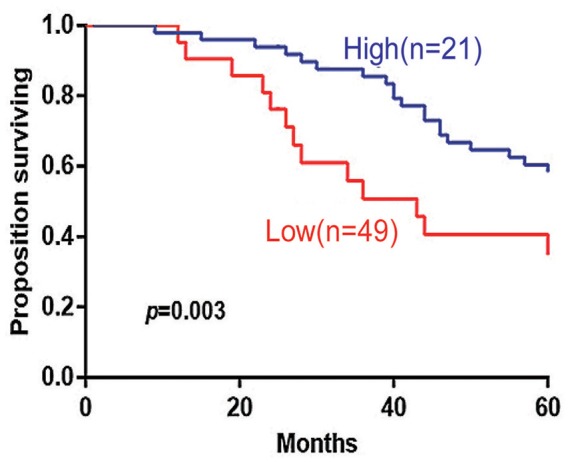
Kaplan-Meier survival curves of high and low 14-3-3beta expression in 70 DLBCL patients.

**Figure 4:**
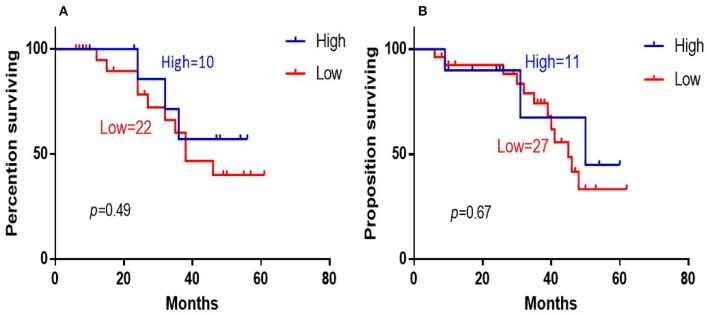
Kaplan-Meier survival curves of 14-3-3beta expression in GCB **(A)** and Non-GCB **(B)** DLBCL patients.

The 5-years overall survival (OS) was determined from the first day of diagnosis to the last day of death or the last day of follow-up. Follow-up data were obtained through telephone interviews and medical records. In survival analyses, low 14-3-3beta expression was significantly associated with adverse overall survival of the DLBCL patients (*P* = 0.003; Figure 3). In GCB and Non-GCB DLBCL patients, there was no significant difference in 5-year OS in the two groups, regardless of low or high expression of 14-3-3beta (*p* > 0.05; Figure 4).

**Figure 5:**
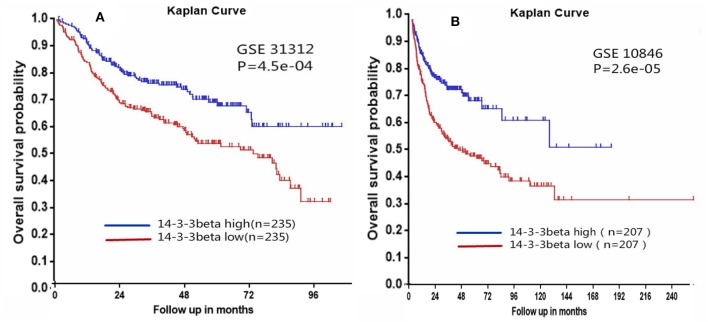
Kaplan-Meier survival curves of low vs. high 14-3-3beta expression in DLBCL patients **(A)** and B cell lymphoma patients **(B)**.

To confirm the 14-3-3beta expression level in DLBCL, we analyzed two independent microarray datasets for B cell lymphoma (GSE10846 and GSE31312) from the National Center for Biotechnology Information Gene Expression Omnibus (GEO) records. The cut-off values for high and low 14-3-3beta expression in the GSE10846 and GSE31312 datasets were 3362.9 and 3147.6, respectively. The correlation of 14-3-3beta expression with prognosis in DLBCL patients was examined based on the difference in survival rate between groups with high and low expression according to the two public B cell lymphoma datasets. Kaplan–Meier analysis indicated that DLBCL patients with low expression of 14-3-3beta had inferior overall survival. In the GSE31312 dataset (*n* = 498), patients with high 14-3-3beta expression had a better overall survival probability than those with low 14-3-3beta expression (*p* = 4.5e-04; Figure 5A). Similarly, in the GSE10846 dataset (*n* = 420), a low level of 14-3-3beta predicted poor survival in B cell lymphoma patients (*p* = 2.6e-05; Figure 5B).

## Discussion

DLBCL is an aggressive malignancy with very heterogeneous genetic abnormalities, clinical features, and response to treatment. This heterogeneity results in highly variable outcomes among patients (14). Although the introduction of rituximab, the 5-year survival rate of patients with DLBCL has significantly improved. The LNH-98.5 trial confirmed that R-CHOP improves outcomes for DLBCL patients (15). In addition, more investigators have provide a great deal of information about DLBCL at the genetic and molecular level, the relationship of therapeutic response and outcome to disease features or heterogeneity for DLBCL pathogenesis still remains a challenge, some patients still relapse after treatment or even progress during treatment.

There are seven distinct isoforms(β, γ, ε, z, η, σ, and τ) that encode seven distinct 14-3-3 proteins in most mammals (16). The 14-3-3 proteins are a ubiquitous family of highly conserved acidic molecules that participate in protein kinase signaling pathways within all eukaryotic cells (17–19) and phosphorylation-dependent protein–protein interactions that regulate multiple cellular functions through the cell cycle, including initiation and maintenance of DNA damage checkpoints, activation of mitogen-activated protein kinases, prevention of apoptosis, proliferation, and malignant transformation (20).

Accumulating evidence indicates increased expression of 14-3-3beta in various types of solider tumors (3–10, 21). In 2018, Seo et al. reported that 14-3-3beta negatively regulated the glioblastoma cells senescence via the ERK-SKP2-p27 pathway (7). In 2016, Tang Y found that 14-3-3β Promotes Migration and Invasion of Human Hepatocellular Carcinoma Cells by Modulating Expression of MMP2 and MMP9 through PI3K/Akt/NF-κB Pathway (22). However, there are relatively few reports on the expression of 14-3-3beta in DLBCL.

In this study, we retrospectively analyzed paraffin slices obtained from 70 DLBCL patients and 50 non-neoplastic lymph nodes (3–5, 8, 10, 12, 21). Immunohistochemical staining showed that 14-3-3beta gene expression was significantly decreased in DLBCL tissues (*P* < 0.0001) compared to that in non-neoplastic lymph nodes. Low 14-3-3beta expression was significantly correlated with extra-nodal status (*P* = 0.026), serum LDH level (*P* = 0.023) and adverse survival of DLBCL patients. In survival analyses, low 14-3-3beta expression was significantly associated with adverse overall survival of the DLBCL patients (*P* = 0.003). Using the Kaplan-Meier analysis module of the R2 microarray analysis and visualization platform (http://r2.amc.nl), we also confirmed that low expression of 14-3-3beta gene had inferior overall survival in DLBCL patients. In summary, these results indicated that DLBCL patients with lower expression of 14-3-3beta have a relatively poor prognosis, which also verifies the conclusions of the data collected above.

However, we analyzed the expression of 14-3-3beta in GCB and Non-GCB DLBCL patients, the results showed there were no statistical difference. Due to the sample size are relatively small, further expansion of the sample size and better study design are needed to confirm the relationship between 14-3-3beta and the occurrence, development and prognosis of DLBCL.

In summary, this is the first study to report the expression of 14-3-3beta gene in DLBCL. There were some limitations in our study. Future experiments are needed to clarify the role of 14-3-3beta gene in DLBCL occurrence and progression. Although the detailed mechanism remains to be explored, this preliminary study suggests that 14-3-3beta gene could be a potential target for DLBCL therapeutics to improve patient outcome and prevent recurrence.

## Data Availability Statement

The datasets generated for this study are available on request to the corresponding author.

## Ethics Statement

The studies involving human participants were reviewed and approved by The First Affiliated Hospital of Zhengzhou University, Zhengzhou, People's Republic of China. Written informed consent to participate in this study was provided by the participants' legal guardian/next of kin.

## Author Contributions

MZ and ZL: supervision. CL: writing-original draft. All authors read the paper and approved the final version.

### Conflict of Interest

The authors declare that the research was conducted in the absence of any commercial or financial relationships that could be construed as a potential conflict of interest.
